# Supplementary material for: Efficacy of traditional Chinese medicine injections for treating idiopathic pulmonary fibrosis: A systematic review and network meta-analysis
Source: PLoS One. 2022 Jul 26;17(7):e0272047. doi: 10.1371/journal.pone.0272047 (PMC9321402; doi:10.1371/journal.pone.0272047)
Supplement: S2 File — (PDF) [file pone.0272047.s002.pdf]

National Institute for Health Research

UNIVERSITY of York
Centre for Reviews and Dissemination

Systematic review

Fields that have an ***asterisk (*)*** *next to them means that they* ***must be answered. Word limits*** *are provided for each section. You will be unable to submit the form if the word limits are exceeded for any section. Registrant means the person filling out the form.*

2. Original language title.

For reviews in languages other than English, give the title in the original language. This will be displayed with the English language title.

3. * Anticipated or actual start date.

Give the date the systematic review started or is expected to start.

03/12/2021

4. * Anticipated completion date.

Give the date by which the review is expected to be completed.

26/01/2022

5. * Stage of review at time of this submission.

This field uses answers to initial screening questions. It cannot be edited until after registration.

Tick the boxes to show which review tasks have been started and which have been completed.

Update this field each time any amendments are made to a published record.

The review has not yet started: No

Review stage	Started	Completed
Preliminary searches	Yes	No
Piloting of the study selection process	No	No
Formal screening of search results against eligibility criteria	No	No
Data extraction	No	No
Risk of bias (quality) assessment	No	No
Data analysis	No	No

Provide any other relevant information about the stage of the review here.

7. * Named contact email.

Give the electronic email address of the named contact.

9. Named contact phone number.

Give the telephone number for the named contact, including international dialling code.

11. * Review team members and their organisational affiliations.

Give the personal details and the organisational affiliations of each member of the review team. Affiliation refers to groups or organisations to which review team members belong. **NOTE: email and country now MUST be entered for each person, unless you are amending a published record.**

Dr Shuaiyang HUANG. Respiratory
Dr Guirui Huang. Beijing University of Chinese Medicine
Dr mingsheng Lv. Beijing University of Chinese Medicine

13. * Conflicts of interest.

List actual or perceived conflicts of interest (financial or academic).

None

14. Collaborators.

Give the name and affiliation of any individuals or organisations who are working on the review but who are not listed as review team members. **NOTE: email and country must be completed for each person, unless you are amending a published record.**

16. * Searches.

State the sources that will be searched (e.g. Medline). Give the search dates, and any restrictions (e.g. language or publication date). Do NOT enter the full search strategy (it may be provided as a link or attachment below.)

ubMed, Web of Science, Embase, The Cochrane Library, MEDLINE, China National Knowledge Infrastructure (CNKI), Wanfang Database, Scientific Journal Database (VIP), China Biology Medicine Database (CBM) were comprehensively searched from inception to November 2021 to identify randomized controlled trials (RCTs) on CMI for treating IPF. Two reviewers independently screened literature, extracted data, and evaluated the risk of bias of included studies. Network meta-analysis was then performed by RevMan 5.4 software and Stata 16.0 software.

18. * Condition or domain being studied.

Give a short description of the disease, condition or healthcare domain being studied in your systematic review.

Idiopathic pulmonary fibrosis (IPF), as a type of pulmonary disease that progresses acutely or slowly into irreversible pulmonary diseases, was caused by various reasons. Healthy tissues are replaced by altered extracellular matrix, and alveolar structure is destroyed, which can lead to reduced lung compliance, interruption of gas exchange, and ultimately respiratory failure and death (Richeldi, Collard, and Jones 2017). IPF is characterized by restrictive ventilatory dysfunction, hypoxemia, and chronic progressive diffuse

pulmonary fibrosis, with clinical symptoms such as progressive dyspnea, wheezing, shortness of breath, and dry cough(Lederer and Martinez 2018). Pirfenidone and nidanib are approved for the treatment of IPF because they can slow down the decline of lung function and disease progression, however, these two drugs have more adverse effects, and no reliable evidence has been seen to confirm that they have significant improvement in patients' symptoms and quality of life, and the cost of both drugs is high, which brings a heavy economic burden to patients and society(Spagnolo et al. 2021).

20. * Intervention(s), exposure(s).

Give full and clear descriptions or definitions of the interventions or the exposures to be reviewed. The preferred format includes details of both inclusion and exclusion criteria.

This study includes 14 CMIs applied in the clinical treatment of IPF, namely Shenfu injection (SF), Shenmai injection (SM), Ligustrazine Injection (LI), Salvia miltiorrhiza polyphenolate injection (SMP), Danhong injection (DH), Xuebijing Injection (XBJ), Shenxiong Injection (SX), Shuxuetong Injection (SXT), Rhodiola injection (RI), Huangqi injection (HQ), safflower yellow sodium chloride injection (HHS), Matrine injection (MI), Shuxuening Injection (SXN), Guanxinning Injection (GXN).

22. * Types of study to be included.

Give details of the study designs (e.g. RCT) that are eligible for inclusion in the review. The preferred format includes both inclusion and exclusion criteria. If there are no restrictions on the types of study, this should be stated.

RCT

24. * Main outcome(s).

Give the pre-specified main (most important) outcomes of the review, including details of how the outcome is defined and measured and when these measurement are made, if these are part of the review inclusion criteria.

Outcomes included the clinical effectiveness rate (CER =Healing rate + markedly effective + effective rate.)

odds ratios (ORs)

26. * Data extraction (selection and coding).

Describe how studies will be selected for inclusion. State what data will be extracted or obtained. State how this will be done and recorded.

Two researchers independently screened the literature and extracted the data according to the screening criteria established. The title and abstract were read first, followed by a full reading of the relevant literature, then cross-checked="checked" value="1" and a third researcher was asked to help judge and discussed the literature where there was disagreement.The data extraction included: (I) Basic information of the included literature (title, first author, date of publication, etc); (II) Basic information of the included patients (number of cases, age, gender, etc); (III) Interventions (drug name, dose, duration of disease, treatment course, etc); (IV) Outcome indicators (clinical efficacy, PaO2, DLco, TGF-?1, etc).

28. * Strategy for data synthesis.

Describe the methods you plan to use to synthesise data. This but should be and describe how the proposed approach will be applied to your data. If meta-analysis is planned, describe the models to be used, methods to explore statistical heterogeneity, and software package to be used.

We performed this study using RevMan (5.4) and Stata (16.0) and constructed a treatment strategy network. (I) For the dichotomous variable, we calculated the odds ratios (ORs), and all of them were expressed with 95% confidence intervals (CIs). When the 95% CI interval does not span 1, the difference between groups is statistically significant; On the contrary, the difference between groups was not statistically significant. (II) For the continuous variables, the mean and the standard deviation of the change amount were calculated based on the mean and standard deviation before and after the treatment, and the mean difference (MD) or standardised mean difference (SMD) was used. Both types of outcomes were presented with their 95% (CIs). When the 95% CIs of the MD or SMD did not include zero, the differences between the groups were considered statistically significant. Then, we used the surface under the cumulative ranking curve (SUCRA) to rank the efficacy of the treatment strategy in each outcome. When SUCRA is closer to 100%, the effectiveness of the intervention under this outcome indicator is greater. (III) Stata software was used to process the outcome data to construct the network diagram of different interventions under the same outcome index.

30. * Type and method of review.

Select the type of review, review method and health area from the lists below.

Type of review

Cost effectiveness
No

Diagnostic
No

Epidemiologic
No

Individual patient data (IPD) meta-analysis
No

Intervention
No

Living systematic review
No

Meta-analysis
No

Methodology
No

Narrative synthesis
No

Network meta-analysis
No

Pre-clinical

No

Prevention
No

Prognostic
No

Prospective meta-analysis (PMA)
No

Review of reviews
No

Service delivery
No

Synthesis of qualitative studies
No

Systematic review
Yes

Other
No

Health area of the review

Alcohol/substance misuse/abuse
No

Blood and immune system
No

Cancer
No

Cardiovascular
No

Care of the elderly
No

Child health
No

Complementary therapies
No

COVID-19
No

Crime and justice
No

Dental
No

Digestive system
No

Ear, nose and throat
No

Education
No

Endocrine and metabolic disorders
No

Eye disorders
No

General interest
No

Genetics
No

Health inequalities/health equity
No

Infections and infestations
No

International development
No

Mental health and behavioural conditions
No

Musculoskeletal
No

Neurological
No

Nursing
No

Obstetrics and gynaecology
No

Oral health
No

Palliative care
No

Perioperative care
No

Physiotherapy
No

Pregnancy and childbirth
No

Public health (including social determinants of health)
No

Rehabilitation
No

Respiratory disorders
Yes

Service delivery
No

Skin disorders
No

Social care
No

Surgery
No

Tropical Medicine
No

Urological
No

Wounds, injuries and accidents
No

Violence and abuse
No

32. * Country.

Select the country in which the review is being carried out. For multi-national collaborations select all the countries involved.

34. Reference and/or URL for published protocol.

If the protocol for this review is published provide details (authors, title and journal details, preferably in Vancouver format)

Add web link to the published protocol.

Or, upload your published protocol here in pdf format. Note that the upload will be publicly accessible.

No I do not make this file publicly available until the review is complete

Please note that the information required in the PROSPERO registration form must be completed in full even if access to a protocol is given.

36. Keywords.

Give words or phrases that best describe the review. Separate keywords with a semicolon or new line. Keywords help PROSPERO users find your review (keywords do not appear in the public record but are included in searches). Be as specific and precise as possible. Avoid acronyms and abbreviations unless these are in wide use.

37. Details of any existing review of the same topic by the same authors.

If you are registering an update of an existing review give details of the earlier versions and include a full bibliographic reference, if available.

38. * Current review status.

Update review status when the review is completed and when it is published.New registrations must be ongoing so this field is not editable for initial submission.
Please provide anticipated publication date

Review_Ongoing

40. Details of final report/publication(s) or preprints if available.

Leave empty until publication details are available OR you have a link to a preprint (NOTE: this field is not editable for initial submission). List authors, title and journal details preferably in Vancouver format.

Give the link to the published review or preprint.
