# Supplementary material for: Efficacy of traditional Chinese medicine injections for treating idiopathic pulmonary fibrosis: A systematic review and network meta-analysis
Source: PLoS One. 2022 Jul 26;17(7):e0272047. doi: 10.1371/journal.pone.0272047 (PMC9321402; doi:10.1371/journal.pone.0272047)
Supplement: S3 File — (DOCX) [file pone.0272047.s003.docx]

**Supplementary file 1**

**Search strategy**

**PubMed:**

(((((((((((((((idiopathic pulmonary fibrosis[MeSH Terms]) OR (idiopathic pulmonary fibrosis[Title/Abstract])) OR (Idiopathic Pulmonary Fibroses[Title/Abstract])) OR (Pulmonary Fibroses[Title/Abstract])) OR (Fibrocystic Pulmonary Dysplasia[Title/Abstract])) OR (Fibrocystic Pulmonary Dysplasias[Title/Abstract])) OR (Cryptogenic Fibrosing Alveolitis[Title/Abstract])) OR (Cryptogenic Fibrosing Alveolitides[Title/Abstract])) OR (Usual Interstitial Pneumonia[Title/Abstract])) OR (Usual Interstitial Pneumonias[Title/Abstract])) OR (Usual Interstitial Pneumonitides[Title/Abstract])) OR (Usual Interstitial Pneumonitis[Title/Abstract])) OR (Familial Idiopathic Pulmonary Fibrosis[Title/Abstract])) AND (((((((((((((((((((((((((((((((((((((((((((((((injections[MeSH Terms]) OR (injections[Title/Abstract])) OR (inject[Title/Abstract])) OR (injectability[Title/Abstract])) OR (injectant[Title/Abstract])) OR (injectants[Title/Abstract])) OR (injectate[Title/Abstract])) OR (injectates[Title/Abstract])) OR (injected[Title/Abstract])) OR (injectible[Title/Abstract])) OR (injectibles[Title/Abstract])) OR (injecting[Title/Abstract])) OR (injectable[Title/Abstract])) OR (Danhong[Title/Abstract])) OR (Reduning[Title/Abstract])) OR (Tanreqing[Title/Abstract])) OR (Safflower yellow[Title/Abstract])) OR (Dazhu rhodiola[Title/Abstract])) OR (Ligustrazine[Title/Abstract])) OR (Shenxiong Glucose[Title/Abstract])) OR (Astragalus[Title/Abstract])) OR (Huangqi[Title/Abstract])) OR (Shenmai[Title/Abstract])) OR (Shenfu[Title/Abstract])) OR (Yanhuning[Title/Abstract])) OR (Shuxuetong[Title/Abstract])) OR (Xuebijing[Title/Abstract])) OR (fisetin[Title/Abstract])) OR (Shengdi[Title/Abstract])) OR (Honghua[Title/Abstract])) OR (Danshen[Title/Abstract])) OR (dihydroartemisinin[Title/Abstract])) OR (Diammonium Glycyrrhizinate[Title/Abstract])) OR (Danggui[Title/Abstract])) OR (Angelica sinensis[Title/Abstract])) OR (Quchongbanjiuju[Title/Abstract])) OR (Xiyanping[Title/Abstract])) OR (matrine[Title/Abstract])) OR (Breviscapine [Title/Abstract])) OR (Dengzhanhuasu[Title/Abstract])) OR (Dengzhanhua[Title/Abstract])) OR (Xinghuayu[Title/Abstract])) OR (Xiangdan[Title/Abstract])) OR (Shengmai[Title/Abstract])) OR (Ciwujia[Title/Abstract])) OR (treatment[Title/Abstract])) OR (therapy[Title/Abstract]))) AND ((((((compare[Title/Abstract]) OR (comparison[Title/Abstract])) OR (comparative[Title/Abstract])) OR (comparing[Title/Abstract])) OR (versus[Title/Abstract])) OR (vs[Title/Abstract]))) AND (((((((Randomized Controlled Trial[Publication Type]) OR (controlled clinical trial[Publication Type])) OR (randomized[Title/Abstract])) OR (randomised[Title/Abstract])) OR (randomly[Title/Abstract])) OR (trial[Title/Abstract])) OR (phase[Title/Abstract]))

**Web of science:**

#5 #4 AND #3 AND #2 AND #1

#4 TS=(Randomized Controlled Trial OR controlled clinical trial OR randomized OR randomised OR randomly OR trial OR phase)

#3 TS=(compare OR comparison OR comparative OR comparing OR versus OR VS)

#2 TS=(treatment OR therapy OR injections OR inject OR injectability OR injectant OR injectants OR injectate OR injectates OR injected OR injectible OR injectibles OR injecting OR injectable OR Danhong OR Reduning OR Tanreqing OR Safflower yellow OR Dazhu rhodiola OR Ligustrazine OR Shenxiong Glucose OR Astragalus OR Huangqi OR Shenmai OR Shenfu OR Yanhuning OR Shuxuetong OR Xuebijing OR fisetin OR Shengdi OR Honghua OR Danshen OR dihydroartemisinin OR Diammonium Glycyrrhizinate OR Danggui OR Angelica sinensis OR Quchongbanjiuju OR Xiyanping OR matrine OR Breviscapine OR Dengzhanhuasu OR Dengzhanhua OR Xinghuayu OR Xiangdan OR Shengmai OR Ciwujia)

#1 TS=(idiopathic pulmonary fibrosis OR Idiopathic Pulmonary Fibroses OR Pulmonary Fibroses OR Fibrocystic Pulmonary Dysplasia OR Fibrocystic Pulmonary Dysplasias OR Cryptogenic Fibrosing Alveolitis OR Cryptogenic Fibrosing Alveolitides OR Usual Interstitial Pneumonia OR Usual Interstitial Pneumonias OR Usual Interstitial Pneumonitides OR Usual Interstitial Pneumonitis OR Familial Idiopathic Pulmonary Fibrosis)

**Embase:**

#5 #1 AND #2 AND #3 AND #4

#4 'randomized controlled trial':it OR 'controlled clinical trial':it OR randomized:ab,ti OR randomised:ab,ti OR randomly:ab,ti OR trial:ab,ti OR phase:ab,ti

#3 compare:ab,ti OR comparison:ab,ti OR comparative:ab,ti OR comparing:ab,ti OR versus:ab,ti OR vs:ab,ti

#2 treatment:ab,ti OR therapy:ab,ti OR injections:ab,ti OR injector:ab,ti OR injectability:ab,ti OR injectant:ab,ti OR injectants:ab,ti OR injectate:ab,ti OR injectates:ab,ti OR injected:ab,ti OR injectible:ab,ti OR injectibles:ab,ti OR injecting:ab,ti OR injectable:ab,ti OR Danhong:ab,ti OR Reduning:ab,ti OR Tanreqing:ab,ti OR 'Safflower yellow':ab,ti OR 'Rhodiola rosea':ab,ti OR Ligustrazine:ab,ti OR 'Shenxiong Glucose':ab,ti OR Astragalus:ab,ti OR Huangqi:ab,ti OR Shenmai:ab,ti OR Shenfu:ab,ti OR Yanhuning:ab,ti OR Shuxuetong:ab,ti OR Xuebijin:ab,ti OR fisetin:ab,ti OR Shengdi:ab,ti OR Honghua:ab,ti OR Danshen:ab,ti OR dihydroartemisinin:ab,ti OR 'Diammonium Glycyrrhizinate':ab,ti OR Danggui:ab,ti OR 'Angelica sinensis':ab,ti OR Quchongbanjiuju:ab,ti OR Xiyanping:ab,ti OR matrine:ab,ti OR Breviscapine:ab,ti OR Dengzhanhuasu:ab,ti OR Dengzhanhua:ab,ti OR Xinghuayu:ab,ti OR Xiangdan:ab,ti OR Shengmai:ab,ti OR Ciwujia:ab,ti

#1 'idiopathic pulmonary fibrosis':ab,ti OR 'Idiopathic Pulmonary Fibroses':ab,ti OR 'Pulmonary Fibroses':ab,ti OR 'Fibrocystic Pulmonary Dysplasia':ab,ti OR 'Fibrocystic Pulmonary Dysplasias':ab,ti OR 'Cryptogenic Fibrosing Alveolitis':ab,ti OR 'Cryptogenic Fibrosing Alveolitides':ab,ti OR 'Usual Interstitial Pneumonia':ab,ti OR 'Usual Interstitial Pneumonias':ab,ti OR 'Usual Interstitial Pneumonitides':ab,ti OR 'Usual Interstitial Pneumonitis':ab,ti OR 'Familial Idiopathic Pulmonary Fibrosis':ab,ti

**The Cochrane Library：**

#1 'idiopathic pulmonary fibrosis':ti,ab,kw OR 'idiopathic pulmonary fibrosis':ti,ab,kw OR 'Idiopathic Pulmonary Fibroses':ti,ab,kw OR 'Pulmonary Fibroses':ti,ab,kw OR 'Fibrocystic Pulmonary Dysplasia':ti,ab,kw OR 'Fibrocystic Pulmonary Dysplasias':ti,ab,kw OR 'Cryptogenic Fibrosing Alveolitis':ti,ab,kw OR 'Cryptogenic Fibrosing Alveolitides':ti,ab,kw OR 'Usual Interstitial Pneumonia':ti,ab,kw OR 'Usual Interstitial Pneumonias':ti,ab,kw OR 'Usual Interstitial Pneumonitides':ti,ab,kw OR 'Usual Interstitial Pneumonitis':ti,ab,kw OR 'Familial Idiopathic Pulmonary Fibrosis':ti,ab,kw

#2 treatment:ti,ab,kw OR therapy:ti,ab,kw OR injections:ti,ab,kw OR injector:ti,ab,kw OR injectability:ti,ab,kw OR injectant:ti,ab,kw OR injectants:ti,ab,kw OR injectate:ti,ab,kw OR injectates:ti,ab,kw OR injected:ti,ab,kw OR injectible:ti,ab,kw OR injectibles:ti,ab,kw OR injecting:ti,ab,kw OR injectable:ti,ab,kw OR Danhong:ti,ab,kw OR Reduning:ti,ab,kw OR Tanreqing:ti,ab,kw OR 'Safflower yellow':ti,ab,kw OR 'Rhodiola rosea':ti,ab,kw OR Ligustrazine:ti,ab,kw OR 'Shenxiong Glucose':ti,ab,kw OR Astragalus:ti,ab,kw OR Huangqi:ti,ab,kw OR Shenmai:ti,ab,kw OR Shenfu:ti,ab,kw OR Yanhuning:ti,ab,kw OR Shuxuetong:ti,ab,kw OR Xuebijin:ti,ab,kw OR fisetin:ti,ab,kw OR Shengdi:ti,ab,kw OR Honghua:ti,ab,kw OR Danshen:ti,ab,kw OR dihydroartemisinin:ti,ab,kw OR 'Diammonium Glycyrrhizinate':ti,ab,kw OR Danggui:ti,ab,kw OR 'Angelica sinensis':ti,ab,kw OR Quchongbanjiuju:ti,ab,kw OR Xiyanping:ti,ab,kw OR matrine:ti,ab,kw OR Breviscapine:ti,ab,kw OR Dengzhanhuasu:ti,ab,kw OR Dengzhanhua:ti,ab,kw OR Xinghuayu:ti,ab,kw OR Xiangdan:ti,ab,kw OR Shengmai:ti,ab,kw OR Ciwujia:ti,ab,kw

#3 compare:ti,ab,kw OR comparison:ti,ab,kw OR comparative:ti,ab,kw OR comparing:ti,ab,kw OR versus:ti,ab,kw OR vs:ti,ab,kw

#4 'Randomized Controlled Trial':pt OR 'controlled clinical trial':pt OR randomized:ti,ab,kw OR randomised:ti,ab,kw OR randomly:ti,ab,kw OR trial:ti,ab,kw OR phase:ti,ab,kw

#5 #1and#2and#3and#4

**Medline：**

S5 S1 AND S2 AND S3 AND S4

S4 AB Randomized Controlled Trial OR AB controlled clinical trial OR AB randomized OR AB randomised OR AB randomly OR AB trial OR AB phase

S3 AB compare OR AB comparison OR AB comparative OR AB comparing OR AB versus OR AB vs

S2 AB treatment OR AB therapy OR AB injections OR AB inject OR AB injectability OR AB injectant OR AB injectants OR AB injectate OR AB injectates OR AB injected OR AB injectible OR AB injectibles OR AB injecting OR AB injectable OR AB Danhong OR AB Reduning OR AB Tanreqing OR AB Safflower yellow OR AB Dazhu rhodiola OR AB Ligustrazine OR AB Shenxiong Glucose OR AB Astragalus OR AB Huangqi OR AB Shenmai OR AB Shenfu OR AB Yanhuning OR AB Shuxuetong OR AB Xuebijing OR AB fisetin OR AB Shengdi OR AB Honghua OR AB Danshen OR AB dihydroartemisinin OR AB Diammonium Glycyrrhizinate OR AB Danggui OR AB Angelica sinensis OR AB Quchongbanjiuju OR AB Xiyanping OR AB matrine OR AB Breviscapine OR AB Dengzhanhuasu OR AB Dengzhanhua OR AB Xinghuayu OR AB Xiangdan OR AB Shengmai OR AB Ciwujia

S1 TI idiopathic pulmonary fibrosis OR TI Idiopathic Pulmonary Fibroses OR TI Pulmonary Fibroses OR TI Fibrocystic Pulmonary Dysplasia OR TI Fibrocystic Pulmonary Dysplasias OR TI Cryptogenic Fibrosing Alveolitis OR TI Cryptogenic Fibrosing Alveolitides OR TI Usual Interstitial Pneumonia OR TI Usual Interstitial Pneumonias OR TI Usual Interstitial Pneumonitides OR TI Usual Interstitial Pneumonitis OR TI Familial Idiopathic Pulmonary Fibrosis

**CNKI:**

(SU=特发性肺纤维化 OR SU=特发性肺间质纤维化 OR SU=肺纤维化 OR SU=肺间质纤维化 OR SU=肺囊性纤维化) AND (SU=注射液 OR SU=注射剂 OR SU=针剂 OR SU=注射 OR SU=注射药 OR SU=丹红 OR SU=热毒宁 OR SU=痰热清 OR SU=红花黄色素 OR SU=大株红景天 OR SU=川芎嗪 OR SU=参芎葡萄糖 OR SU=黄芪 OR SU=参麦 OR SU=参附 OR SU=炎琥宁 OR SU=疏血通 OR SU=血必净 OR SU=漆黄素 OR SU=生地 OR SU=红花 OR SU=丹参 OR SU=双氢青蒿素 OR SU=甘利欣 OR SU=当归 OR SU=浓当归 OR SU=驱虫斑鸠菊 OR SU=喜炎平 OR SU=苦参碱 OR SU=灯盏花素 OR SU=灯盏花 OR SU=杏花雨 OR SU=香丹 OR SU=生脉 OR SU=刺五加) AND FT=随机

**Wanfang Database:**

主题:(特发性肺纤维化 or 特发性肺间质纤维化 or 肺纤维化 or 肺间质纤维化 or 肺囊性纤维化) and 主题:(注射液 or 注射剂 or 针剂 or 注射 or 注射药 or 丹红 or 热毒宁 or 痰热清 or 红花黄色素 or 大株红景天 or 川芎嗪 or 参芎葡萄糖 or 黄芪 or 参麦 or 参附 or 炎琥宁 or 疏血通 or 血必净 or 漆黄素 or 生地 or 红花 or 丹参 or 双氢青蒿素 or 甘利欣 or 当归 or 浓当归 or 驱虫斑鸠菊 or 喜炎平 or 苦参碱 or 灯盏花素 or 灯盏花 or 杏花雨 or 香丹 or 生脉 or 刺五加) and全部:(随机)

**Scientific Journal Database (VIP):**

M=(特发性肺纤维化 OR 特发性肺间质纤维化 OR 肺纤维化 OR 肺间质纤维化 OR 肺囊性纤维化) AND M=(注射液 OR 注射剂 OR 针剂 OR 注射 OR 注射药 OR 丹红 OR 热毒宁 OR 痰热清 OR 红花黄色素 OR 大株红景天 OR 川芎嗪 OR 参芎葡萄糖 OR 黄芪 OR 参麦 OR 参附 OR 炎琥宁 OR 疏血通 OR 血必净 OR 漆黄素 OR 生地 OR 红花 OR 丹参 OR 双氢青蒿素 OR 甘利欣 OR 当归 OR 浓当归 OR 驱虫斑鸠菊 OR 喜炎平 OR 苦参碱 OR 灯盏花素 OR 灯盏花 OR 杏花雨 OR 香丹 OR 生脉 OR 刺五加) AND U=随机

**CBM:**

(特发性肺纤维化[标题] OR 特发性肺间质纤维化[标题] OR 肺纤维化[标题] OR 肺间质纤维化[标题] OR 肺囊性纤维化[标题]) AND (注射液[摘要] OR 注射剂[摘要] OR 针剂[摘要] OR 注射[摘要] OR 注射药[摘要] OR 丹红[标题] OR 热毒宁[摘要] OR 痰热清[摘要] OR 红花黄色素[摘要] OR 大株红景天[摘要] OR 川芎嗪[摘要] OR 参芎葡萄糖[摘要] OR 黄芪[摘要] OR 参麦[摘要] OR 参附[摘要] OR 炎琥宁[摘要] OR 疏血通[摘要] OR 血必净[摘要] OR 漆黄素[摘要] OR 生地[摘要] OR 红花[摘要] OR 丹参[摘要] OR 双氢青蒿素[摘要] OR 甘利欣[摘要] OR 当归[摘要] OR 浓当归[摘要] OR 驱虫斑鸠菊[摘要] OR 喜炎平[摘要] OR 苦参碱[摘要] OR 灯盏花素[摘要] OR 灯盏花[摘要] OR 杏花雨[摘要] OR 香丹[摘要] OR 生脉[摘要] OR 刺五加[摘要]) AND (随机[全部字段])
