# Supplementary material for: Efficacy of traditional Chinese medicine injections for treating idiopathic pulmonary fibrosis: A systematic review and network meta-analysis
Source: PLoS One. 2022 Jul 26;17(7):e0272047. doi: 10.1371/journal.pone.0272047 (PMC9321402; doi:10.1371/journal.pone.0272047)
Supplement: S4 File — (DOCX) [file pone.0272047.s004.docx]

**Abbreviations**

CMIs: Chinese medicine injections; CER: Clinical effectiveness rate; DH: Danhong injection; DLCO: Diffusing capacity of the lungs for carbon monoxide; FVC: Forced Vital Capacity; FEV1%: Forced Expiratory Volume 1%; FEV1/FVC%; Forced Expiratory Volume 1/ Forced Vital Capacity; GXN: Guanxinning Injection; HQ: Huangqi injection; HHS: Safflower yellow sodium chloride injection; IIIC: Type III collagen; IPF: Idiopathic pulmonary fibrosis; LI: Ligustrazine Injection; MI: Matrine injection; Network meta-analysis (NMA); ORs: odd ratios; PaCO_2_: Carbon dioxide partial pressure; PaO_2_: Oxygen partial pressure; RCTs: Randomized controlled trials; RI: Rhodiola injection; SF: Shenfu injection; SM: Shenmai injection; SMP: Salvia miltiorrhiza polyphenolate injection; SX: Shenxiong Injection; SXT: Shuxuetong Injection; SXN: Shuxuening Injection; SUCRA: surface under cumulative ranking curve; TLC: Total lung capacity; TGF: Transforming growth factor; TCM: Traditional Chinese medicine; WM: Western medicine; XBJ: Xuebijing Injection.
