# Supplementary material for: Efficacy of traditional Chinese medicine injections for treating idiopathic pulmonary fibrosis: A systematic review and network meta-analysis
Source: PLoS One. 2022 Jul 26;17(7):e0272047. doi: 10.1371/journal.pone.0272047 (PMC9321402; doi:10.1371/journal.pone.0272047)
Supplement: S1 Table — (DOCX) [file pone.0272047.s005.docx]

**Characteristics of the included studies.**

| Study ID | N (E/C) | SEX (M/F) | AGE (YERS) (E/C) | I (E) | I (C) | Cs (day) | Outcome |
| --- | --- | --- | --- | --- | --- | --- | --- |
| Yanlei et al.(2012) | 46/46 | - | - | DH+WM | WM | 120 | ⑨ |
| Ping et al.(2014) | 45/45 | 52/38 | 50.2±(9.6)/ 49.5±(8.7) | DH+WM | WM | 84 | ②④⑩ |
| Yonghua et al.(2013) | 38/38 | 46/30 | 48.86±(8.14) | DH+WM | WM | 42 | ①②④⑩ |
| Xuming et al.(2015) | 30/30 | 31/19 | 69.1/65.8 | DH+WM | WM | 42 | ②④ |
| Ping et al.(2015) | 34/34 | 50/18 | 52.3±(2.3)/ 53.5±(2.0) | DH+WM | WM | 84 | ②④⑩ |
| Baoping et al.(2018) | 30/30 | 37/23 | 61.56±(12.33)/ 61.80±(12.18) | DH+WM | WM | 84 | ②④⑨ |
| Shiqi et al.(2011) | 41/41 | 39/43 | 52/55 | DH+WM | WM | 42 | ①②④ |
| Jianxi et al.(2012) | 50/51 | 55/46 | 55.31±(9,36)/ 54.96±(9.72) | DH+WM | WM | 56 | ① |
| Xiazhou et al.(2016) | 27/27 | 37/17 | 55.4±(5.6) | DH+WM | WM | 120 | ②⑨ |
| Li et al.(2016) | 40/40 | 53/27 | 62.3±(8.9)/ 62.8±(8.7) | DH+WM | WM | 84 | ③④⑧⑨⑩ |
| Minying et al.(2019) | 60/60 | 71/49 | 3.91±(1.24)/ 3.87±(1.13) | DH+WM | WM | 90 | ⑤⑥⑦⑨ |
| Honglian et al.(2016) | 59/58 | 76/41 | 59.63±(3.25)/ 59.65±(3.29) | DH+WM | WM | 120 | ⑨ |
| Yun et al.(2015) | 35/32 | 37/30 | 48.2±(16.3)/ 50.8±(17.4) | DH+WM | WM | 84 | ① |
| Yuan et al.(2020) | 60/60 | 54/66 | 64±(5.7)/ 62±(6.2) | DH+WM | WM | 84 | ②③④⑤⑥⑦⑨⑩ |
| Fei et al.(2016) | 25/25 | 27/23 | 35~68/36~67 | DH+WM | WM | 42 | ①②④ |
| Wei et al.(2014) | 42/42 | 51/33 | 36~70.5/37~70 | DH+WM | WM | 42 | ①②④⑩ |
| Yuxia et al.(2016) | 50/50 | 72/28 | 67.3±(4.2)/ 66.8±(5.1) | DH+WM | WM | 42 | ①②④ |
| Siyu et al.(2021) | 27/27 | 29/25 | 68.89±(0.21)/ 68.91±(0.18) | DH+WM | WM | - | ②④ |
| Fengping et al.(2012) | 24/24 | 29/19 | 50.63±(10.63)/ 50.86±(10.22) | DH+WM | WM | 42 | ①②④⑩ |
| Yafa et al.(2016) | 35/35 | 40/30 | 62.41±(9.82)/ 61.53±(12.52) | DH+WM | WM | 84 | ②④⑧⑨ |
| Hongmei et al.(2012) | 34/34 | - | 56.32±(3.29)/ 56.38±(3.27) | DH+WM | WM | 56 | ①②④ |
| Xiuhe et al.(1998) | 20/18 | 22/16 | 58.8±(11.6)/  64.9±(12.2) | LI+WM | WM | 60 | ②⑤⑦ |
| Fengqi et al.(2003) | 26/26 | 27/25 | 54.17±(12.3)/  52.15±(11.3) | LI+WM | WM | 56 | ① |
| Dongjie et al.(2012) | 30/30 | 33/27 | 55.16±(12.38)/51.48±(13.61) | LI+WM | WM | 56 | ②⑤⑧ |
| Shupei et al.(2010) | 19/20 | 23/16 | 68~76 | LI+WM | WM | 365 | ① |
| Yanling et al.(2010) | 30/30 | 29/31 | 62/64 | LI+WM | WM | 84 | ①②④⑤⑥⑧ |
| Gonggui et al.(2013) | 50/50 | 59/41 | 44.3±(3.7) | LI+WM | WM | 84 | ①④⑤⑥ |
| Jianxi et al.(2013) | 52/50 | 51/51 | 53.12±(10.7)/ 54.48±(9.8) | LI+WM | WM | 56 | ①②⑤⑦ |
| Miao et al.(2016) | 25/24 | 27/22 | 68.5 | LI+WM | WM | 28 | ①②④⑤⑥ |
| Xianping et al.(2012) | 30/31 | 35/25 | 56.47±(9.56)/ 55.83±(9.70) | LI+WM | WM | 14 | ① |
| Xiuzhi.  (2004) | 18/15 | 24/9 | 54.18±(11.3)/ 53.15±(12.2) | LI+WM | WM | 56 | ① |
| Yong et al.(2015) | 35/35 | 57/13 | 55.8±(12.9)/ 53.6±(11.7) | LI+WM | WM | 84 | ①②③④⑤⑥⑦⑧ |
| Wangping et al.(2009) | 56/30 | 54/32 | 39.8 | LI+WM | WM | 77 | ① |
| Zhenguo et al.(2013) | 20/18 | 23/15 | 63.2/61.55 | LI+WM | WM | 21 | ① |
| Hechen et al.(2009) | 33/33 | 39/27 | 55.16±(12.38)/49.48±(13.61) | LI+WM | WM | 84 | ① |
| Ti et al.(2013) | 16/16 | 16/16 | 42~70 | LI+WM | WM | 14 | ①②③ |
| Linlin et al.(2019) | 49/49 | 53/45 | 38.5±(2.3)/ 38.4±(2.1） | SX+WM | WM | 168 | ①②③⑤⑦⑩ |
| Lili et al.(2011) | 39/18 | - | 40~70 | SX+WM | WM | 90 | ①④⑤⑦ |
| Linlin et al.(2019) | 41/40 | 48/33 | 67.1±(3.0)/ 66.7±(2.4) | SX+WM | WM | 56 | ①②③⑦⑧⑩ |
| Shibo et al.(2011) | 23/25 | 31/17 | 56±(13)/ 58±(12) | SX+WM | WM | 21 | ②④⑧ |
| Danyang et al.(2010) | 56/30 | 54/31 | 39.8 | SX+WM | WM | 80 | ① |
| Yanxia et al.(2020) | 44/44 | 46/42 | 50.81±(1.64)/ 49.83±(1.38) | SX+WM | WM | 15 | ①②③⑤⑦ |
| Lei et al.(2011) | 40/20 | 33/27 | 55.9±(4.2)/ 56.1±(4.1) | SX+WM | WM | 70 | ⑩ |
| Yanling et al.(2012) | 20/15 | 22/13 | 54.32±(13.44)/ 52.15±(11.35) | SXT+WM | WM | 15 | ①②⑤⑦ |
| Jing et al.(2016) | 35/35 | 51/19 | 45.9±(3.5)/ 46.1±(3.6) | SXT+WM | WM | 84 | ①②③④⑤⑦⑧ |
| Yu et al.(2015) | 30/30 | 41/19 | 54.8±(7.3)/ 53.9±(8.1) | SXT+WM | WM | 84 | ①②③④⑤⑦⑧ |
| Limin et al.(2015) | 21/17 | 20/18 | 71.23±(10.31)/ 69.52±(12.61) | XBJ+WM | WM | 28 | ① |
| Liming et al.(2013) | 18/18 | 20/16 | 71±(9)/  65±(7) | XBJ+WM | WM | 7 | ① |
| Ruirui et al.(2010) | 32/32 | 39/25 | 58.6 | XBJ+WM | WM | 14 | ①②③④⑥ |
| Gang et al.(2012) | 23/23 | 29/17 | 56 | HQ+WM | WM | 90 | ① |
| Gaozhong et al.(2010) | 26/21 | 30/17 | 58/57 | HQ+WM | WM | 180 | ①②④⑤⑥ |
| Xiaomei et al.(2020) | 42/42 | 51/33 | 60.42±(6.59)/ 60.18±(6.43) | HQ+WM | WM | 14 | ① |
| Qifang et al.(2011) | 20/20 | 22/18 | 58±(6)/  57±(6) | MI+WM | WM | 28 | ⑤⑦ |
| Li et al.(2011) | 31/25 | 31/25 | 53.4 | MI+WM | WM | 84 | ① |
| Weihua et al.(2013) | 19/19 | 21/17 | 34.72±(8.02)/ 33.5±(10.04) | MI+WM | WM | 14 | ⑩ |
| Yuanying et al.(2013) | 24/24 | 27/21 | 60.1±(7.62)/ 61.24±(7.29) | HHS+WM | WM | 90 | ⑤⑦ |
| Guixiang et al.(2016) | 35/35 | 47/23 | 62.41±(9.82)/ 61.53±(12.52) | HHS+WM | WM | 84 | ②④⑧⑨ |
| Xun et al.(2015) | 10/10 | 12/8 | 61.1(4.5) | SF+WM | WM | 28 | ②③④⑤⑩ |
| Zhendong et al.(2016) | 31/30 | 41/20 | 59.24±(3.56)/ 62.94±(3.92) | SM+WM | WM | 180 | ①④⑥ |
| Xingyu et al.(2012) | 32/33 | 34/31 | 45~81/  46~79 | SMP+WM | WM | 14 | ②④⑥ |
| Jie et al.(2014) | 20/20 | - | 18~65 | RI+WM | WM | 72 | ①②④⑤ |
| Ming et al.(2010) | 60/60 | 54/46 | 64.5±(9.0)/ 65.4±(8.2) | SXN+WM | WM | 14 | ①②③⑤ |
| Xiangmin et al.(2007) | 60/42 | 88/14 | 45~81/  46~80 | GXN+WM | WM | 28 | ① |

**Note:** ①CER; ②PaO_2_; ③PaCO_2_; ④DLCO; ⑤FVC; ⑥TLC; ⑦FEV1%; ⑧FEV1/FVC%; ⑨IIIC; ⑩TGF.

**Abbreviations:** C, control group; E, experimental group; M, male; F, female; N, number; I, intervention; Cs, course.
